# My Back Exercise app: an automated exercise intervention supported by educational notifications, a sleep programme and diet advice to improve function in people with chronic non-specific low back pain – protocol for a superiority, adaptive multi-arm multi-stage randomised controlled trial

**DOI:** 10.1136/bmjopen-2024-098324

**Published:** 2025-08-01

**Authors:** Anelise Moreti Cabral Silveira, Josielli Comachio, Carlos Mesa-Castrillon, Katharine Roberts, Paula Beckenkamp, Emma K Ho, Manuela L Ferreira, Christopher J Gordon, Serigne N Lo, Kim L Bennell, Rachel K Nelligan, Rowena J Field, Paulo Ferreira

**Affiliations:** 1 Faculty of Medicine and Health, Sydney Musculoskeletal Health, The University of Sydney, Sydney, New South Wales, Australia; 2 Musculoskeletal Research Hub, Charles Perkins Centre, The University of Sydney, Sydney, New South Wales, Australia; 3 The George Institute for Global Health, UNSW Sydney, Sydney, New South Wales, Australia; 4 Medicine & Health, University of New South Wales, Sydney, New South Wales, Australia; 5 Faculty of Medicine, Health and Human Sciences, Macquarie University, Sydney, New South Wales, Australia; 6 CIRUS, Centre for Sleep and Chronobiology, Woolcock Institute of Medical Research, Sydney, New South Wales, Australia; 7 Faculty of Medicine and Health, The University of Sydney, Sydney, New South Wales, Australia; 8 Melanoma Institute Australia, The University of Sydney, Sydney, New South Wales, Australia; 9 Charles Perkins Centre, The University of Sydney, Sydney, New South Wales, Australia; 10 Centre for Health, Exercise and Sports Medicine, Department of Physiotherapy, School of Health Sciences, The University of Melbourne, Melbourne, Victoria, Australia

**Keywords:** Spine, Back pain, Exercise, eHealth, Chronic Pain

## Abstract

**Introduction:**

As chronic low back pain (LBP) remains one of the most pressing global health challenges, digital health emerges as an opportunity to deliver evidence-based care at scale. In this context, the My Back Exercise app has been developed to support people with LBP in self-managing their condition. This study aims to determine the effectiveness of the My Back Exercise app to improve physical function in people with chronic non-specific LBP.

**Methods and analysis:**

A single-blind, superiority, adaptive randomised controlled trial encompassing a multi-arm multi-stage design will be conducted and reported according to the Standard Protocol Items: Recommendations for Interventional Trials (SPIRIT), Template for the Intervention Description and Replication for telehealth (TIDieR-telehealth), and the Consolidated Standards of Reporting Trials (CONSORT) Extension for Adaptive Designs. Informed consent will be obtained from all participants. Eligible participants will be randomly assigned in a 1:1:1:1:1 ratio to either the control group, receiving education alone, or one of four intervention groups ((1) education, notifications and exercise modules; (2) education, notifications, exercise and sleep modules; (3) education, notifications, exercise and diet modules; and (4) education, notifications, exercise, sleep and diet modules) through the app. The study consists of two stages with the possibility of dropping arms for futility using pre-specified decision rules at the end of the first stage. A total of 370 participants aged 18 years or older, with chronic non-specific LBP, will be recruited Australia-wide from the general community. The primary outcome will be self-reported physical function measured by the Patient-Specific Functional Scale at 6 weeks post-randomisation. Data will be analysed using the Student’s t-test or the non-parametric Mann-Whitney U test.

**Ethics and dissemination:**

This trial was approved by the Human Research Ethics Committee (HREC) of The University of Sydney (HREC Approval No. 2023/HE000772). The findings of this study will be disseminated via scientific publications, reports and conference presentations, and strategically disseminated to the wider community and relevant policymakers.

**Trial registration number:**

Australian New Zealand Clinical Trials Registry (ACTRN12624000319572).

**STRENGTHS AND LIMITATIONS OF THIS STUDY**

- This is a single-blind, superiority, adaptive randomised controlled trial encompassing a multi-arm, multi-stage design, with the possibility of dropping arms for futility.
- The mobile application delivers a tailored exercise programme, supplemented by education, sleep and diet modules, in addition to self-management push notifications.
- The trial has broad eligibility criteria to maximise external validity and applicability of study findings to people with chronic back pain.
- Limitations include the lack of blinding of study participants, due to the nature of the interventions.
- Barriers related to Internet access and digital literacy may impact recruitment, attrition rates and adherence to the intervention.

## Introduction

### Background and rationale

 Chronic musculoskeletal conditions pose major global health challenges to individuals, society and governments.^1^ Low back pain (LBP), especially, has prevailed as the leading cause of years lived with disability worldwide for nearly three decades.^2 3^ In addition to overloading healthcare systems,^4^ LBP imposes a substantial economic burden at both individual and societal levels,^5 6^ largely due to high levels of healthcare utilisation and productivity loss.^7^,^10^ Despite extensive efforts to prevent LBP and improve its management, only a slight decrease in prevalence rates has been observed over the last 30 years, and the burden associated with the condition continues to expand in the face of a growing and ageing population.^11^ This scenario is further exacerbated by under-resourced health systems and workforce shortages,^2^ which, in turn, represent underlying barriers to healthcare access and uptake of evidence-based interventions.^11^,^14^ Altogether, these factors prefigure an escalating public health challenge.^15 16^

Timely access to first-line, evidence-based healthcare remains a major obstacle to universal health coverage in both high- and low-income countries,^17^ particularly when considering people residing in remote areas.^18^ In Australia, remoteness contributes to workforce shortages^19 20^ and an increased likelihood of patients reporting barriers to accessing healthcare professionals.^20^ In 2017, the ratio of physiotherapists to the population in Australia substantially decreased with increasing levels of geographic remoteness, with only 42 physiotherapists per 100 000 population in very remote areas, compared with 90 physiotherapists per 100 000 population in major cities.^20^ A similar pattern has been reported in the USA, with 44 physiotherapists per 100 000 population in rural areas, compared with 65 physiotherapists per 100 000 population in urban areas.^21^

Meanwhile, the digital revolution has been shaping healthcare systems through technological breakthroughs and innovation.^22 23^ In this context, digital health has emerged as a promising strategy to support patients in overcoming barriers to accessing evidence-based healthcare.^24^ Digital health is a broad umbrella term that describes the use of information and communication technologies to support health.^24^ The strategic use of digital health solutions has become particularly attractive in the field of musculoskeletal care due to their capabilities to deliver comprehensive care and potentially enhance the efficiency, affordability and cost-effectiveness of healthcare.^25^ Furthermore, exercise and self-management strategies, considered core components in the rehabilitation journey for musculoskeletal disorders, are feasible approaches for remote delivery.

Evidence of successful deployment of digital interventions to promote musculoskeletal health has been demonstrated in high-quality randomised controlled trials (RCTs), including the EMPoweR^26^ and My Knee Exercise^27^ studies. The EMPoweR trial demonstrated that a patient-tailored eHealth physiotherapy intervention offers greater and clinically worthwhile improvements for physical function, measured by the Patient-Specific Functional Scale (PSFS), compared with usual care for people with chronic LBP.^26^ However, physiotherapists were required to remotely deliver synchronous interventions via eHealth, which could incur costs to patients and reliance on healthcare providers. On the other hand, the My Knee Exercise trial demonstrated the effectiveness of a web-based, self-directed strengthening exercise and physical activity programme to improve knee pain and function in people with knee osteoarthritis.^27^ Although there is preliminary evidence that exercise programmes delivered through automated digital platforms (ie, with no need for a clinician interface) can offer small benefits for people with LBP (eg, selfBACK platform in Norway),^28^ no previous studies have investigated the automated delivery of a patient-centred exercise programme specifically targeting patients’ individual needs to improve their physical function. Moreover, to date, uncertainty still exists regarding the effectiveness and cost-effectiveness of digital strategies, especially mobile applications (ie, apps), to manage chronic LBP.^29^

In this context, the My Back Exercise app was developed to support people with LBP in self-managing their condition. Conceptual and empirical frameworks for the app were instigated through collaborative discussions with the My Knee Exercise^27^ and the EMPoweR^26^ study teams. The app was co-designed by clinical researchers and healthcare professionals, considering the best available evidence. Consumer representatives, clinicians and experts in the field provided input in terms of the content, design and usability of the app. Complete details of the app development process were published elsewhere.^30 31^ Self-directed and automated, the mobile application delivers tailored exercises to improve patient-specific physical function based on the best available evidence on the management of chronic LBP.^32^ The app is automated (no clinician interface), dynamic (progressive and goal-oriented, based on patients’ feedback) and supported by push notifications.^33^,^35^ Additionally, the app includes education, sleep and diet modules to support LBP self-management comprehensively.

RCTs are considered the gold standard method for evaluating the effect of health interventions. Nevertheless, they intrinsically incur high costs and constraints, including long study duration and large sample sizes.^36^ Master protocol frameworks^37^ and adaptive designs^36 38 39^ have been proposed to overcome the shortcomings associated with conventional RCTs. While embracing the underlying complexity of healthcare research,^40^ these innovative approaches provide the landscape to flexibly, comprehensively and adaptively evaluate a broad spectrum of hypotheses under the same overarching infrastructure,^37 38^ improving the efficiency of clinical trials. Particularly in adaptive designs, accumulated trial data are used to implement preplanned modifications while the trial is still ongoing.^36 38 39^ These adaptations can potentially lead to reduced, yet appropriate, sample sizes to answer multiple research questions and/or reduced study duration, therefore optimising the use of resources, reducing costs and streamlining the pipeline from research to clinical practice.^36 38^ They are also considered more ethical in some instances, as they have the potential to minimise the number of participants exposed to ineffective treatments.^41^ Although increasingly adopted in medical research, adaptive approaches are still underused in musculoskeletal health and rehabilitation research.^40^ To evaluate the My Back Exercise app, we propose an adaptive trial encompassing a multi-arm multi-stage (MAMS) design. This approach is particularly advantageous when multiple promising interventions are evaluated,^42 43^ as it allows the early dropping of study arms that are shown to be futile in accordance with prespecified decision rules.^38 41^

### Objectives

The purpose of this study will be to determine, through an adaptive MAMS RCT, whether the My Back Exercise app, consisting of a 6-week, automated, tailored exercise intervention, standard LBP education, educational push notifications, a sleep educational programme and diet advice, compared with a control intervention, consisting of app-based standard LBP education, is effective for improving physical function in people with chronic non-specific LBP at 6 weeks after randomisation (ie, immediate post-intervention). In addition, this study aims to identify which combination of components within the My Back Exercise app is the most effective for eliciting improvement in physical function. The secondary aim of this study will be to determine whether the My Back Exercise app is effective for improving secondary outcomes when compared with app-based standard LBP education for people with chronic non-specific LBP. Secondary outcomes will include pain intensity, pain frequency, disability, health-related quality of life, sleep quality, consumption of highly processed food, exercise importance, exercise self-efficacy, adherence to exercise recommendations, co-intervention use, health literacy level, global perceived effect, impression of change, overall satisfaction, usefulness of the app and frequency of the push notifications. Additionally, data will be collected on process measures (ie, conversion rate, recruitment rate and rate of completed follow-up), the smallest worthwhile effect and adverse events.

## Methods and analysis

### Patient and public involvement

Researchers, clinicians, consumers and our partner, Musculoskeletal Health Australia, were involved in the design and usability testing of the My Back Exercise app.^31^ Musculoskeletal Health Australia is the consumer organisation working with and advocating on behalf of more than 7 million Australians living with musculoskeletal conditions. Musculoskeletal Health Australia has been involved in all stages of the development of the mobile application, endorses it, and will be one of the partners for recruitment. The organisation will also play an essential role in strategically disseminating the study results to the wider community, aiming to increase consumers’ awareness of evidence-based recommendations arising from the trial. Ultimately, Musculoskeletal Health Australia will also facilitate the implementation of the trial’s outcomes into health systems.

### Trial design and setting

This study will be a parallel, superiority, decentralised, adaptive RCT encompassing a MAMS design with individual randomisation in a 1:1:1:1:1 ratio. The trial protocol has been designed according to the Standard Protocol Items: Recommendations for Interventional Trials (SPIRIT) statement,^44^ and the interventions are reported according to the TIDieR-telehealth checklist,^45^ an extension of the Template for the Intervention Description and Replication (TIDieR) checklist. The trial will be conducted and reported according to the Adaptive Designs CONSORT Extension (ACE) statement,^46^ an extension of the Consolidated Standards of Reporting Trials (CONSORT) statement.

The clinical trial will be conducted entirely online in a community setting in Australia. Recruitment, consent, eligibility assessment, enrolment, intervention delivery and data collection procedures will be performed remotely using digital technology. The intervention will be delivered via an app that participants will download via the app store according to their smartphone or tablet operating system (ie, Apple App Store or Google Play Store).

### Eligibility criteria

Consenting participants will receive a phone call from a trained member of the research team, who will complete the Eligibility Screening Form over the phone and determine eligibility. Data will be entered directly into the Research Electronic Data Capture (REDCap). The phone call will also be essential to verify the authenticity of responses and ensure the individuals are genuine participants. To be included in the study, participants will need to meet all the inclusion criteria and none of the exclusion criteria.

### Inclusion criteria

- Aged 18 years or older.
- Report an episode of non-specific LBP of at least 12 weeks duration, with or without leg pain. LBP is defined as pain on the posterior aspect of the body from the lower margin of the twelfth ribs to the lower gluteal folds, with or without referred pain in one or both lower limbs.^47^ Non-specific LBP is defined as LBP without a specific cause diagnosed, and the absence of serious spinal pathology or indicators of potentially serious conditions (‘red’ flags).
- Had LBP diagnosed by a healthcare practitioner.
- Have a smartphone or tablet with an Internet connection.
- Have independent mobility and eyesight to see the app content and exercise independently and safely (ie, not regularly using mobility aids).
- Have a sufficient understanding of English.

### Exclusion criteria

- Known or suspected serious spinal pathology (eg, fracture, inflammatory disorder); specific diagnosis of LBP (eg, sciatica, spinal stenosis grade 3–4); self-reported radicular symptoms (eg, reflex changes, motor loss).
- Spinal surgery in the past 12 months.
- LBP caused by involvement in a road traffic crash in the last 12 months or currently receiving ongoing litigation.
- Fibromyalgia or systemic/inflammatory condition(s) that are not controlled (eg, systemic lupus erythematosus, multiple sclerosis).
- Comorbid health condition(s) diagnosed by a medical practitioner that would prevent participation in physical activity or exercise programmes (eg, chronic heart conditions).

Additional Exercise and Physical Activity Screening Questions will be applied and considered on a case-by-case basis to determine if medical clearance for participation in an unsupervised, home-based exercise programme is required before enrolment. In that case, the participant will be given a Medical Clearance Referral Form to be completed by a medical practitioner (eg, general practitioner).

### Intervention and comparator

### Intervention components

### Exercise module (‘Exercises’)

The exercise module comprises a 6-week, automated, home-based, tailored exercise programme, generated based on the participant’s self-reported functional level, and involving a graded approach to progression in intensity and complexity. The content of the exercise module has been designed specifically for this study, considering the best available evidence, and incorporating frameworks such as health coaching,^48 49^ motivational communication,^49 50^ cognitive behavioural therapy^51^ and self-management approaches^32 52^ to facilitate attributes such as empowerment, self-confidence, autonomy and self-efficacy.^52^,^55^

On registering an account within the app, participants will be asked to complete a modified version of the PSFS,^56^ which will be used to determine the type and difficulty level of the exercises suggested for them. The modified version of the PSFS consists of a list of 22 predetermined tasks, from which the participants will be asked to identify three important activities they are unable to perform or have difficulty with because of their LBP. The list of activities covers the most commonly reported by the participants of the EMPoweR clinical trial^26^ and includes an additional option for other tasks not specified in the list. The participant will be asked to rate their ability to perform each activity on an 11-point numerical rating scale ranging from 0 (unable to perform the activity) to 10 (able to perform the activity). The activities selected and the scores given for each activity will guide the type and difficulty level of the exercises suggested by the app. For each activity, participants with scores under 4 will be allocated to the beginner exercise level, scores between 4 and 7 to the intermediate exercise level and scores over 7 to the advanced exercise level. The thresholds were established based on 1119 observations from the EMPoweR RCT. The app will suggest an exercise programme focused on improving strength and mobility, with up to three exercises, and three additional options for swapping if the participant wishes to replace a particular exercise with another one. The additional exercise will also be targeted at the same PSFS activities selected by the participant. For each exercise, there will be a video demonstration, as well as verbal and written instructions on how to set up the workout space, position the body properly and perform the exercise. In addition, participants are provided with safety tips (eg, choosing appropriate surfaces to stand or hold on to and avoid trips and falls) and suggestions for adaptations that might become necessary to relieve pain or discomfort. Participants who experience persistent discomfort or worsening of symptoms associated with a given exercise will be instructed to use the ‘swap exercise’ feature of the app to receive an alternative option. When the additional exercise is still not suitable, participants will be encouraged to reduce the dose (eg, number of sets and repetitions) and/or the range of motion, and gradually build up as tolerated. If symptoms persist or worsen, participants will be advised to discontinue that specific exercise until they receive a new weekly programme. The exercises will involve body weight only, although some may require minimal equipment (eg, a mat, chair, table, step, pillow, handrail or wall for support). Examples of strengthening exercises include squats, lunges, push-ups and curl-ups. Examples of flexibility exercises include trunk, hamstrings, piriformis and quadriceps stretching.

The dosage of the resistance training according to difficulty level was established based on the American College of Sports Medicine^57 58^ and the International Exercise Recommendations in Older Adults.^59^ The International Exercise Recommendations in Older Adults^59^ was used, given that the prevalence of LBP has been shown to increase with age, peaking at approximately 85 years of age, as highlighted in the latest report of the Global Burden of Disease Report.^14^ A total of 8–12 repetitions will be recommended for isotonic exercises, 10–30 s hold for isometric exercises and 10–30 s hold for flexibility exercises; one to three sets; three times a week; at an exercise intensity of seven to nine in all programmes, as rated on an 11-point scale of rating of perceived exertion (RPE).^60^ Each exercise session is estimated to last between 20 and 30 min.

A graded approach to progression in intensity and complexity of the exercises will be adopted. Intensity is increased by changing body position. At the end of each week, the app will prompt participants to rate their exertion for each exercise on the RPE scale.^60^ The exercises rated with an RPE of six or lower will be replaced by new exercises to add variety and increase the exercise challenge. The exercises rated with an RPE of seven or higher will continue in the exercise programme for another week.

Participants will be recommended to complete their exercise programme at least 3 days per week across the 6-week exercise programme, totalling at least 18 exercise sessions. There will be no distinction among intervention groups. Considering that the exercise programme should be performed three times a week and that each exercise session should take approximately 20–30 min, completing the exercise module should take approximately 60–90 min per week for 6 weeks.

### Education module (‘Back Tips’)

The education module consists of a 6-week intervention containing written information and audio tracks designed specifically for this study, considering the best available evidence,^15 16 32 48 49 51 61^ and is complemented with links to online, readily available resources. The education module provides information about non-specific LBP, including the nature of LBP, symptoms, evidence-based treatments, pain management and other frequently asked questions about LBP. This module also contains information and guidance to assist participants in increasing their general physical activity level. Information includes why and how to increase general physical activity, how to track and safely increase daily steps, activity pacing and how to design a physical activity plan. Additionally, this module provides sleep and diet information related to LBP. Every week, new content will be made available (ie, unlocked) and participants will be recommended to complete the weekly content at their convenience. Completing the education module should take approximately 10–30 min per week for 6 weeks.

### Sleep module (‘Sleep Tips’)

The sleep module consists of a 4-week tailored sleep educational programme containing written information and audio tracks designed specifically for this study, with advice from expert researchers in the sleep field, considering the best available evidence.^62 63^ The sleep module provides information about sleeping habits, sleep efficiency and how sleep and LBP are connected. Participants will learn how they can optimise their sleep, and they will be given hints, tips and personalised goals on how to improve their sleeping habits through breathing and relaxation training and stimulus control strategies. Once a week, participants will be asked to review sleep information and select a goal for the week. Based on the goal selected, participants will receive daily or weekly reminders to work on their goal. The reminder will be a push notification at an appropriate time of the day (ie, 08:00 for a daytime tip and 20:00 for an evening tip). Completing the sleep module should take approximately 10–15 min per week for 4 weeks.

### Diet module (‘Diet Tips’)

The diet module consists of a 6-week intervention containing written information and audio tracks designed specifically for this study, considering the best available evidence,^64^,^66^ and is complemented with links to online, readily available resources. The diet module provides information about the basics of nutrition, including food groups, ultra-processed foods, the impact of sugar on health and the role of antioxidants. Participants will be offered weekly tasks on how to read nutrition labels, make healthier food choices and use a healthy diet as a coping strategy to self-manage their chronic pain. The content is strictly educational, encouraging participants to adopt healthier eating habits. No recommendations will be made regarding specific diets, and no meal plans or recipes to follow will be provided. Every week, new content will be made available (ie, unlocked) and participants will be recommended to complete the weekly content at their convenience. Completing the diet module should take approximately 10–30 min per week for 6 weeks.

### Automated push notifications

Participants will receive lifestyle-based, self-management push notifications providing encouragement, advice and motivation. The content will cover education about LBP, and tips about exercise, physical activity, sleep, diet, mood and use of care and medication, aiming at improving self-care and pain coping strategies, as well as promoting engagement with the app. Each notification will be approximately 160 characters in length and will include the name of the app. Some will also include the participant’s preferred contact name. Participants will receive up to four notifications per week for 6 weeks in random time slots at 09:00, 12:30, 16:00 and 18:00. There will be no distinction among intervention groups.

The content of the push notifications has been adapted from the TEXT4MyBACK trial,^34^ where a bank of text messages was developed for people with LBP. The messages were co-designed by consumer representatives from Musculoskeletal Health Australia, multidisciplinary clinicians and experts, considering the best available evidence,^1516 32 67^,^71^ and incorporating concepts from the behaviour change model,^72^ as described elsewhere.^34^ Key domains were defined based on the ‘Managing Your Pain: An A-Z Guide’,^73^ developed and published by Musculoskeletal Health Australia. The frequency of the push notifications was also established based on previous research.^34 74^

### Study arms

**Figure 1:**
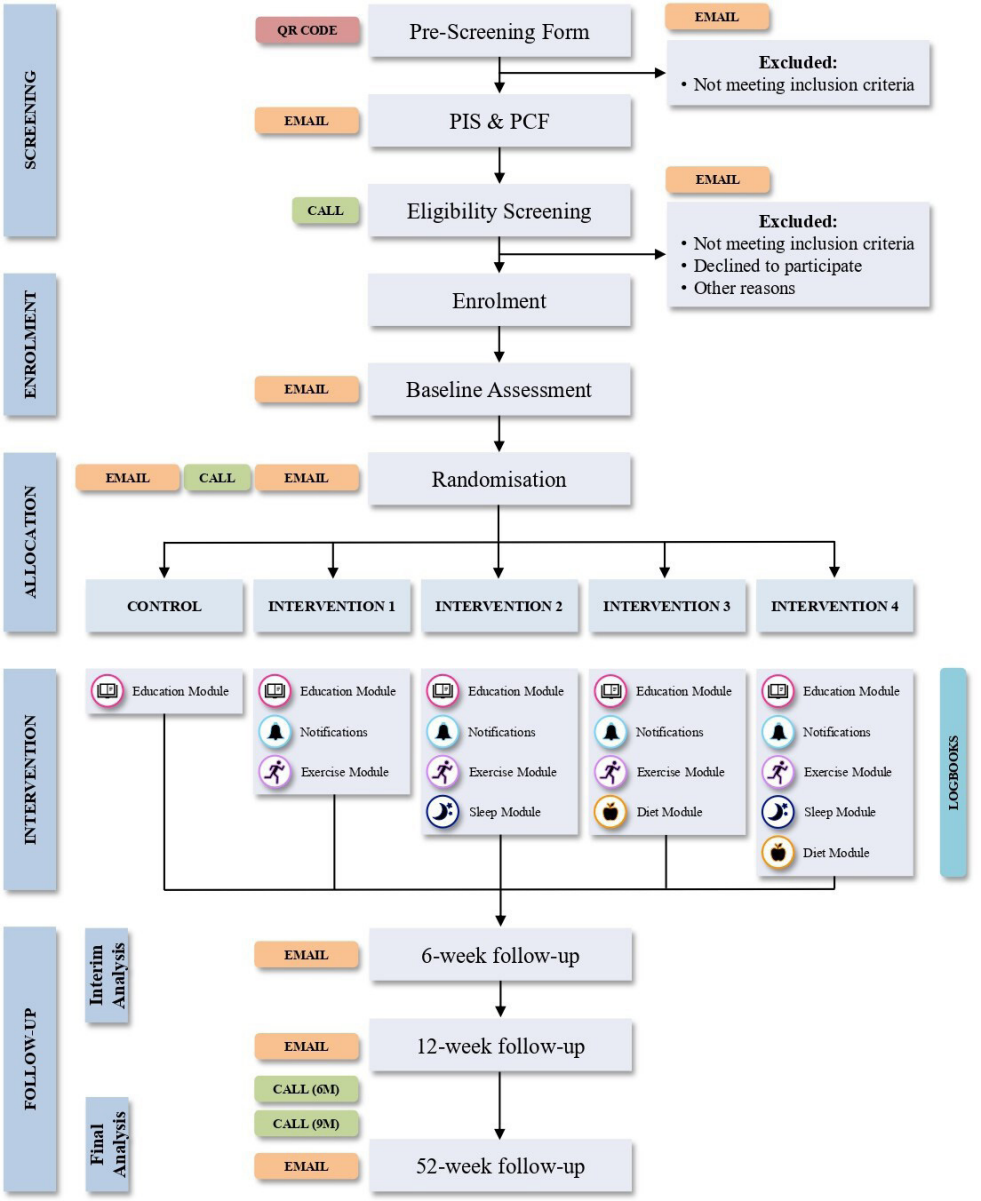
Consolidated Standards of Reporting Trials flow chart. PCF, Participant Consent Form; PIS, Participant Information Statement; QR, quick response.

This MAMS trial will comprise five study arms, including one control group and four intervention groups. All participants will receive access to the My Back Exercise app. However, access to specific components within the app will be available according to the participant’s allocation, as described next and illustrated in figure 1. Recruitment and enrolment will commence at the same time for all study arms, and there will be no introduction of new arms into the study. No specific recommendations will be made regarding concomitant care during the trial.

- Control group: education module alone.
- Intervention group 1: education module + educational notifications + exercise module.
- Intervention group 2: education module + educational notifications + exercise module + sleep module.
- Intervention group 3: education module + educational notifications + exercise module + diet module.
- Intervention group 4: education module + educational notifications + exercise module + sleep module + diet module.

All participants will have access to specific components of the app (according to their group allocation) during the entire study (ie, 1 year after the last recruited participant completes the intervention period). After this period, offline access to the full content of the app will be offered to all participants in the study for an additional 6 weeks.

### Monitoring adherence to the intervention

To measure adherence to the intervention, participants will complete a self-reported activity tracker within the My Back Exercise app for the exercise and sleep modules (which offer tailored content), and a weekly electronic logbook via REDCap for the education and diet modules. The invitation to complete the weekly electronic logbook will be sent via scheduled and automated emails via REDCap. Additionally, app use and engagement (eg, frequency of use, time spent, content accessed) will be collected in a de-identified format using data analytics, drawn from the app’s backend system via the Matomo web analytics platform.^75^ At 6 and 9 months after randomisation, there will also be a brief phone call with the participants to follow-up on adherence and engagement with the app. Adherence will be calculated for each study arm and the entire study.

For the exercise module, participants will be recommended to complete at least three exercise sessions per week, totalling 18 sessions over the 6-week exercise programme. Data will be drawn primarily from the app’s self-reported daily activity tracker, and additional insights will be provided by data analytics from Matomo.^75^ Adherence levels will be assessed based on the proportion of self-reported completed exercise sessions relative to the number of recommended sessions and categorised as low (0%–35%, or up to six exercise sessions completed), moderate (35%–70%, or 7–12 exercise sessions completed) and high (70%–100%, or 13–18 exercise sessions completed). Adherence will be considered acceptable when participants complete at least 70% of their prescribed exercise programme.^76^

For the sleep module, participants may choose weekly or daily goals over the 4-week sleep programme. Data will be drawn primarily from the app’s self-reported daily or weekly activity tracker according to the goal selected by the participant, and additional insights will be provided by data analytics from Matomo.^75^ Adherence levels will be assessed based on the proportion of self-reported completed goals relative to the number of recommended goals and categorised as low (0%–35%), moderate (35%–70%) and high (70%–100%). Adherence will be considered acceptable when participants complete at least 70% of their prescribed sleep programme.

For the education and diet components, participants will be recommended six structured modules over the 6-week app programme. Data will be drawn primarily from the weekly electronic logbooks, categorised as ‘not started’, ‘partially completed’ or ‘completed’, and additional insights will be provided by data analytics from Matomo.^75^ Adherence levels will be assessed based on the number of self-reported completed modules and categorised as low (0%–35%, or up to two modules completed), moderate (35%–70%, or three to four modules completed) and high (70%–100%, or five to six modules completed). Adherence will be considered acceptable when participants complete at least 70% of their prescribed education or diet programmes.

A planned exploratory analysis will be conducted to investigate the potential impact of adherence on the magnitude of the treatment effects.

### Outcomes

#### Primary outcome

The primary outcome measure will be self-reported physical function, measured by the PSFS^56 77^ at baseline, 6, 12 and 52 weeks after randomisation. The primary time-point is 6 weeks after randomisation (ie, immediate post-intervention), while the secondary time-points are 12 and 52 weeks after randomisation. The PSFS, originally proposed by Stratford *et al*,^77^ is a valid, reliable and responsive self-report instrument^56^ designed to assess functional change in people with musculoskeletal disorders. Participants identify three important activities they are unable to do or have difficulty with because of their LBP and rate their ability to perform each activity. Each activity is rated on an 11-point numerical rating scale ranging from 0=‘unable to perform activity’ to 10=‘able to perform the activity at preinjury level’. The total score, calculated by summing the numerical ratings for each activity, is provided on a discrete scale ranging from 0 to 30, where higher scores indicate better function.

#### Secondary outcomes

Secondary outcome measures will be grouped into domains and will include:

Symptoms and function:

- Pain intensity, measured by the Numeric Rating Scale (NRS)^78^ at baseline, 6, 12 and 52 weeks after randomisation.
- Pain frequency, measured by a self-reported question at baseline, 6, 12 and 52 weeks after randomisation.
- Disability, measured by the Roland-Morris Disability Questionnaire (RMDQ)^56 79^ at baseline, 6, 12 and 52 weeks after randomisation.

Quality of life and general health:

- Health-related quality of life (HRQoL), measured by the EuroQol 5-Dimension 5-Level (EQ-5D-5L)^80^ at baseline, 6, 12 and 52 weeks after randomisation.
- Sleep quality, measured by the Pittsburgh Sleep Quality Index (PSQI)^81 82^ at baseline, 6, 12 and 52 weeks after randomisation.
- Consumption of highly processed food, measured by the Short Screening Questionnaire of Highly Processed Food Consumption (sQ-HPF)^64^ at baseline, 6, 12 and 52 weeks after randomisation.

Health behaviours and beliefs:

- Exercise importance, measured by a self-reported question at baseline and 6 weeks after randomisation. Participants indicate how important it is to them to do regular exercise to manage their back condition. Answer options are presented on a 7-point Likert-type scale ranging from 1=‘not at all important’ to 7=‘extremely important’, where higher scores indicate higher importance.
- Exercise self-efficacy, measured by the Self-Efficacy for Exercise (SEE) Scale^83^ at baseline, 6, 12 and 52 weeks after randomisation.
- Adherence to exercise recommendations, measured by the Exercise Adherence Rating Scale (EARS)^84^ at 6 weeks after randomisation.

Healthcare utilisation and engagement:

- Co-intervention use, measured by a self-reported questionnaire specifically designed for this study at baseline, 6, 12 and 52 weeks after randomisation.
- Health literacy level, measured by the Health Literacy Questionnaire (HLQ)^85^ at baseline, 6, 12 and 52 weeks after randomisation.

Patient perception of change:

- Global perceived effect, measured by the Global Perceived Effect (GPE) scale^86^ at 6, 12 and 52 weeks after randomisation.
- Impression of change in overall status, measured by the Patient’s Global Impression of Change (PGIC)^87^ at 6, 12 and 52 weeks after randomisation.

Satisfaction and usability of the app:

- Overall satisfaction, measured by a self-reported question at 6 weeks after randomisation. Participants indicate how satisfied they are with the electronic resources they accessed as part of this study. The question is rated on a 7-point Likert-type scale ranging from 1=‘very dissatisfied’ to 7=‘very satisfied’, where higher scores indicate higher satisfaction.
- Usefulness of the app, measured by self-reported questions at 6 and 52 weeks after randomisation. Participants will be asked to rate how useful they found the My Back Exercise app, overall and in each module. Answer options are presented on a 7-point Likert-type scale ranging from 1=‘completely useless’ to 7=‘extremely useful’, where higher scores indicate the app or the modules were perceived to be more useful.
- Frequency of the push notifications, measured by self-reported questions at 6 and 52 weeks after randomisation in the intervention groups only. Participants indicate whether the number of notifications received over the past 6 weeks was the right amount for them. Answer options are presented on a 7-point Likert-type scale ranging from 1=‘strongly disagree’ to 7=‘strongly agree’, where higher scores indicate higher agreement. Participants who select ≤3 will be further asked whether they received 1=‘too few’ or 2=‘too many’.

#### Process measures

- Conversion rate, measured using data analytics (ie, audit of study-specific screening and enrolment logs) at baseline. The number and percentage of participants screened who proceed to enrol in the clinical trial will be calculated.
- Recruitment rate, measured using data analytics (ie, audit of study-specific screening and enrolment logs) at 6 weeks after randomisation. The average number and percentages of participants enrolled in the study per month and per day of active recruitment will be calculated.
- Rate of completed follow-up, measured using data analytics (ie, audit of study-specific database) at 6, 12 and 52 weeks after randomisation. The number and percentage of participants enrolled in the study who completed the follow-up assessment will be calculated.

#### Other measures

- Smallest Worthwhile Effect^88^ in function, measured at baseline. Participants will be asked to nominate the smallest score in the PSFS (total and for each activity) they would need to achieve to consider the costs, risks and inconveniences of using a mobile application intervention worthwhile.
- Adverse events, collected through a self-reported form at 2, 4, 6 and 52 weeks after randomisation.

### Data collection methods

After providing informed consent and meeting the eligibility criteria, participants will be enrolled in the study and assigned a unique study identification code, which will be documented in the participants’ records to protect their privacy. After that, they will be invited to complete the baseline assessment. The baseline assessment will include questions on sociodemographic information (eg, date of birth, sex, location, language used at home, education level, current employment status), anthropometric characteristics (eg, weight, height and body mass index), smoking status, comorbid conditions, LBP history (duration, characteristics, pattern of symptoms, surgical history), use of corticosteroids in the past month and the study outcomes.

Baseline and follow-up data collection and management will be performed online via REDCap, using standardised surveys designed to collect data directly from participants via a secure webpage. REDCap is an online, encrypted, customisable data capture tool, hosted by the University of Sydney.^89 90^ Patient-reported outcomes will be collected using valid and reliable self-reported questionnaires (detailed in the Outcomes section). To ensure the completeness of data, participants will receive reminders either via email, SMS or phone call, prompting them to complete a given questionnaire. To minimise missing data, the research team will encourage participants to complete, at a minimum, the primary outcome assessment at the 6-week follow-up assessment. If necessary, completion over the telephone will be made available.

Participants may choose to withdraw at any point. Participants may also choose to stop accessing the app while continuing with some or all aspects of data collection. If a participant withdraws from the study, the nature, timing and reasons for withdrawal will be recorded, and any data provided up to the point of the participant’s withdrawal will be kept in the study records and will be used in statistical analyses, unless the participant specifically requests to withdraw all their data from the study. In this case, their data will be withdrawn, and no attempt will be made to impute missing values for statistical analyses.

### Harms

An adverse event is defined as any undesirable, unfavourable or unintended sign, symptom or disease experienced by a participant in the course of clinical research, which does not necessarily have a causal relationship with the study interventions. A serious adverse event is defined as an unforeseen medical event that occurs in the course of clinical research that results in the participant’s death, is life-threatening to the participant, requires the inpatient hospitalisation or prolongation of existing hospitalisation, or leads to the participant having a persistent or significant disability/incapacity.^91^ This study has been designed to minimise or prevent potential risks. Nevertheless, adverse events are expected and may include flare-ups of LBP, exercise-induced muscle pain, soreness and cramps, unexpected trips and falls, and additional treatments sought for LBP.

Fortnightly, participants will be prompted by email to report the occurrence of adverse events through an Adverse Events Report Form via REDCap during the 6 weeks of intervention and, again, at 52 weeks after randomisation. Participants will indicate any changes in their condition, and the research team will evaluate whether it was related to the intervention or not. When appropriate, participants will be asked to provide further information on whether the adverse event persisted for more than 24 hours and whether medical attention was sought. Participants will be encouraged to contact the research team if they have ongoing or unresolved concerns about the adverse event and its relationship with the study interventions. The research team will monitor reported adverse events until their resolution. Potential harms resulting from participation in the study will not incur any special compensation arrangements. The type of adverse event and the proportions of participants experiencing each event will be reported.

Serious adverse events will be recorded and reported to the Human Research Ethics Committee (HREC) and study sponsor, in accordance with relevant regulatory standards.

### Participant timeline

The study flow chart and study stages are shown in figures1  2, respectively. Study visits and procedures schedule are detailed in table 1. Recruitment has commenced in June 2024.

**Figure 2:**
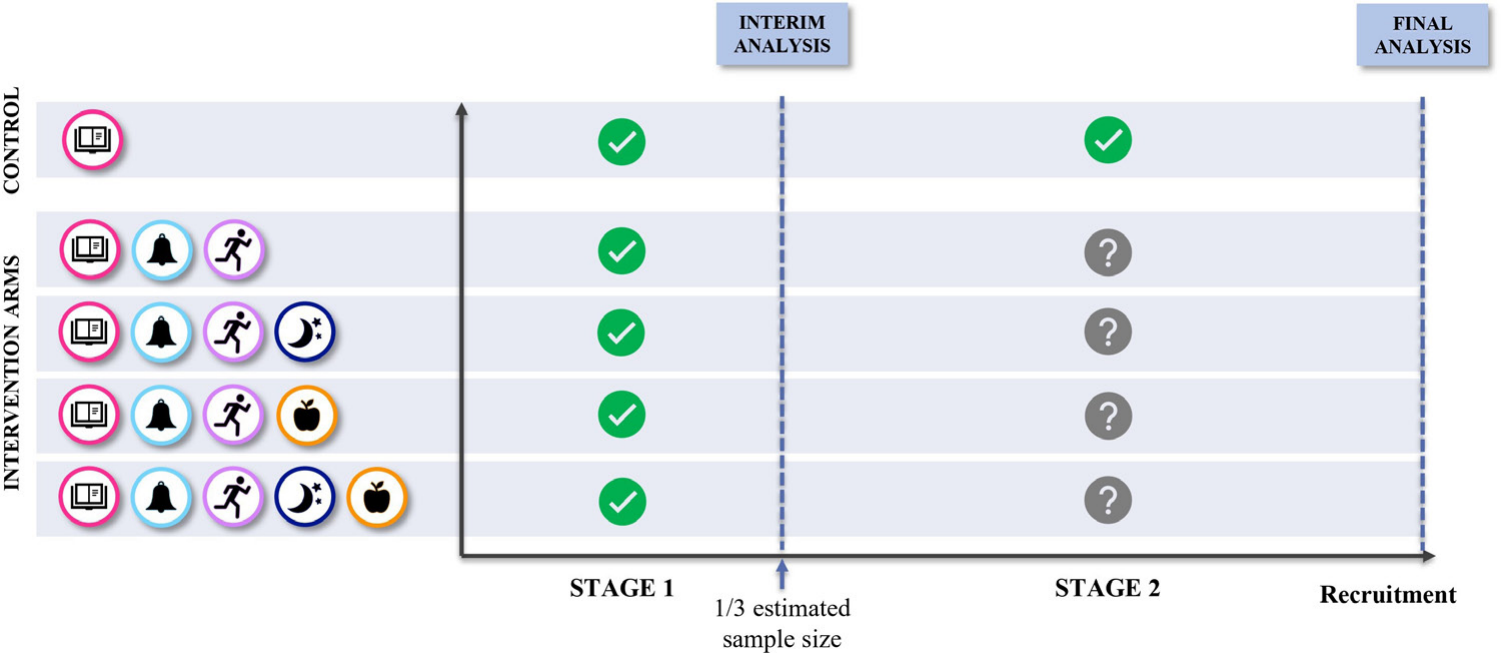
Multi-arm multi-stage design.

**Table 1:** Study visits and procedures schedule

Procedures	Study period and timepoints						
	Screening and Enrolment	Baseline	Allocation	Intervention	Follow-up data collection		
	Week −4 to −1	Week 0	Week 0	Week 1 to 6	Week 6	Week 12	Week 52
Screening and Enrolment							
Informed consent	X						
Eligibility screening	X						
Baseline assessment		X					
Allocation			X				
Interventions							
Control group			X	X	X	X	X
Intervention groups			X	X	X	X	X
Measures							
General characteristics		X					
Primary outcome							
Physical function		X			X	X	X
Secondary outcomes							
Pain intensity		X			X	X	X
Pain frequency		X			X	X	X
Disability		X			X	X	X
Quality of life		X			X	X	X
Sleep quality		X			X	X	X
Consumption of HPF		X			X	X	X
Exercise importance		X			X		
Exercise self-efficacy		X			X	X	X
Adherence to exercise					X		
Co-intervention use		X			X	X	X
Health literacy level		X			X	X	X
Global perceived effect					X	X	X
Impression of change					X	X	X
Overall satisfaction					X		
Usefulness of the app					X		X
Push notifications frequency					X		X
Process measures							
Conversion rate		X					
Recruitment rate					X		
Rate of completed follow-up					X	X	X
Other measures							
Smallest worthwhile effect		X					
Adverse events				X	X		X

X indicates the timepoint(s) when each study procedure will occur.

HPF, highly processed food.

### Sample size

The sample size calculation was conducted a priori, considering a MAMS design with five arms (one control arm and four intervention arms) and two stages that consist of one interim and the final analysis. The primary endpoint was established at 6 weeks post-randomisation (ie, immediate post-intervention). A smallest worthwhile effect on the PSFS score (0–30) of 4 points was chosen to estimate the sample size, considering the literature^76^ and the nature of the intervention (ie, a 6-week digital intervention with no face-to-face interaction).

The sample size calculation was based on a two-sided type I error of 5% (significance level), a power of 80% to control for a type II error and an SD of 5.8 using a generalisation of the power requirements for testing multiple active arms to one control group, as proposed by Dunnett.^92^ The calculation resulted in 21 participants per arm at the first stage and 42 participants per arm at the second stage, reaching a total sample size of 315 participants. Allowing for a loss to follow-up rate of 15% at 6 weeks, the study would require a total sample size of 370 participants (n=25 in the first stage and 49 in the second stage per group). This total sample represents the maximum number of participants that will be enrolled when all the intervention arms meet the criteria for being maintained through the second interim stage (ie, no arm is dropped after the interim analysis). Therefore, there is the possibility that the study terminates with a smaller sample size if, at the interim analysis, one or more intervention arms meet the criteria for being dropped. For instance, if one, two or three arms are dropped after the interim, the study sample will decrease to 321, 272 and 223, respectively.

### Recruitment

Potential participants will be recruited Australia-wide from the community via advertisements published in, but not limited to, internet-based news outlets and newsletters, seniors card newsletters, community lists and social media including Facebook, Instagram, X (formerly known as Twitter) and YouTube. In addition, the George Institute’s Join-Us Research Participant Register (https://www.joinus.org.au/), a secure online register that matches participants with research studies in Australia, will be used.

The advertisements will contain a link or quick response code to the Pre-Screening Questionnaire, available on REDCap, where potential participants can register their interest in the study. REDCap is an online, encrypted, customisable data capture tool, hosted by the University of Sydney. The welcome page will be secured by a Google reCAPTCHA feature, enabled to protect the public survey from abuse by ‘bots’ and prevent automated software programs from submitting fraudulent responses. After passing the reCAPTCHA test, participants will have access to the welcome page and the Pre-Screening Questionnaire.

The Pre-Screening Questionnaire aims to identify potentially eligible participants and collect contact details of individuals interested in the study. Once identified, potentially eligible participants will receive an email with a link to the online Participant Information Statement, containing detailed information about the study and the research team, as well as the contact details for the study, and the Participant Consent Form, both available on REDCap.

The digital divide was an additional layer of complexity that was carefully considered from an equity and inclusivity perspective when designing the study. In the Australian context, critical barriers to digital inclusion include uneven access to networks and digital infrastructure, affordability of devices and data, and a lack of digital literacy and ability, as measured by the Australian Digital Inclusion Index (ADII).^93^ In 2017, Australia was one of the leading adopters of smartphones globally, with 88% of Australians owning one.^94^ In 2023, 99% of Australian adults had Internet access, with 95% of online adults connecting mostly over their mobile phones.^95^ Finally, the 2023 Australian National ADII score was 73.2, with 14.2% and 9.4% Australians classified as digitally excluded and highly digitally excluded, respectively.^93^ Although we do not envisage that a large proportion of the population will be automatically excluded from participating in this study due to digital inequalities, we recognise that this may pose a limitation of the study, with potential impact on recruitment and attrition rates, as well as adherence to the intervention.

### Randomisation: sequence generation, allocation concealment mechanism and implementation

After completing the baseline assessment, participants will be randomly allocated to either the control group or one of the four intervention groups. Computer-generated randomisation will be prepared in permuted blocks of sizes 10–15. To conceal allocation, the randomisation schedule will be generated via a computer, password-protected software by a statistician not involved in participant recruitment, scheduling or assessment. The person who will determine if a potential participant is eligible for inclusion in the trial will be unaware, when this decision is made, to which group the participant will be allocated.

All participants will receive access to the ‘My Back Exercise’ app. However, access to specific intervention components within the app will be available according to the participant’s group allocation, as described next and illustrated in figure 1:

- Control group: education module alone.
- Intervention group 1: education module + educational notifications + exercise module.
- Intervention group 2: education module + educational notifications + exercise module + sleep module.
- Intervention group 3: education module + educational notifications + exercise module + diet module.
- Intervention group 4: education module + educational notifications + exercise module + sleep module + diet module.

After randomisation, participants will receive an email containing detailed instructions on how to download, instal and access the app. This will be followed by a phone call from a member of the research team to help with these procedures if needed, ensure proper functioning of the app and confirm participants only have access to the app components they have been randomly allocated to. Once participants download and instal the app on their own devices, they will receive a welcome email prompting them to access the app and commence the recommended intervention programme. This time point will mark the start of the 6-week intervention programme.

### Blinding

Participants and specific members of the research team (ie, the members of the Data and Safety Monitoring Board (DSMB)) will be unblinded to group allocation. All other research team members, including data collectors conducting baseline and follow-up assessments if required, and data analysts, will remain blinded to group allocation.

As data collection over the telephone will be made available, if necessary, the effectiveness of blinding arrangements will be evaluated at the end of the intervention by asking the assessors to guess the arm to which the participant belonged. Although strategies will be implemented to prevent premature unblinding, breaking of blinding during the study should be expected when this is essential to ensure participant safety. Premature unblinding will be promptly documented and communicated to the study sponsor. The participant will be included in the intention-to-treat analysis in all analyses.

### Data management

All data collection will be performed via REDCap, a secure web application for building and managing online surveys and databases, supported by the University of Sydney. To promote data quality, data collection instruments will incorporate built-in validation features, including branching logic, range checks and required fields, where applicable. Pilot testing of the surveys will be conducted before implementation to ensure proper functioning. In addition, the research team will conduct regular data audits and scrutiny to ensure data quality, integrity, accuracy and completeness. When identified, inconsistencies, omissions or errors will be explored and resolved.

Participant data within the app will be uploaded to the Amazon Web Services, a secure server hosted by Trade eXpansion, the app development team. Appropriate security methods will be implemented to protect the data. Credentials with the right permissions are required, and multi-factor authentication is mandatory. The name of the participant is stored locally on the participant’s phone and is not uploaded to the server.

Digital study data, associated records and study documentation, including confidential data collected from participants, will be exported and stored on the Research Data Store, a password-protected research data storage infrastructure hosted and managed by the University of Sydney, in a re-identifiable (coded) format. Once data collection is completed, identifiers will be removed from the dataset to protect participants’ privacy. Appropriate security methods will be implemented to protect the data by password-protected encryption for digital files.

### Statistical methods

All statistical analyses will be conducted following the intention-to-treat principle. Baseline characteristics will be summarised by arm using descriptive statistics and reported with measures of frequency (proportion), mean, SD and 95% CI. Differences in dichotomous variables will be expressed as risk difference and number needed to treat or number needed to harm with 95% CI. Continuous variables will be summarised and reported with mean and SD in case of a Gaussian (normal) distribution or as the median and IQR in case of a skewed distribution.

At the interim and final analysis, differences in outcome data between each intervention arm and the control group will be analysed using the Student’s t-test in the case of a Gaussian (normal) distribution. Otherwise, the non-parametric Mann-Whitney U test will be used after correcting for baseline differences, if required. Changes in health-related quality of life scores will be analysed using the Wilcoxon test. The Cox proportional hazards regression modelling will be used to adjust for potential confounding factors. For all analyses, statistical significance will be indicated by a p value <0.05.

A planned hypothesis-generating (exploratory) subgroup analysis will be conducted considering diet and sleep. Interaction models will compare the effectiveness of the sleep module in people with low and high scores on the PSQI, as well as the diet module in people with low and high scores on the sQ-HPF. Additionally, a planned exploratory analysis will be conducted to investigate the potential impact of adherence on the magnitude of the treatment effects.

The interim analysis will be conducted after completion of the 6-week intervention programme by the first 25 participants in each arm. Whether and how the trial will continue will be determined by the outcome of the interim analysis, which will allow dropping intervention arms that are deemed futile, in accordance with the following prespecified decision rules considering the primary endpoint (ie, change in mean PSFS score from baseline to 6 weeks after randomisation):

- If the change in an intervention arm is no more than 0.5 points greater than the control group, this intervention arm will be dropped. Under this rule, all four intervention arms may be dropped, and the trial terminated. This decision rule was established based on previous adaptive trials investigating conservative interventions in the field of musculoskeletal health.^96^
- If the change in one intervention arm is more than 4 points higher than the other, the worst-performing intervention arm will be dropped. This decision rule was established considering the smallest worthwhile effect on disability, reported in previous studies as a 15% incremental improvement beyond the natural recovery.^97^ Natural recovery, in turn, is considered a 30% improvement from baseline. Based on our previous study (EMPoweR), a 45% improvement from baseline would correspond to a 5.4-point between-group difference. For this study, this effect size was downgraded to 4 points due to the nature and duration of the intervention (ie, a 6-week digital intervention with no face-to-face interaction).

The decision rules were carefully designed to minimise the risk of prematurely and incorrectly dropping promising treatment arms, potentially leading to a failure to reject the null hypothesis (type II error), while ensuring that the preplanned adaptations would still guarantee increased trial efficiency. Two complementary decision rules were proposed to account for the comparison between each treatment arm against the control group (first rule) and the pairwise comparison among all treatment arms (second rule). Both decision rules are intended to work as indicators of futility, therefore configuring a ‘drop-the-loser’ design that also allows early stopping for futility. Early stopping for efficacy was not planned in this trial.

### Data monitoring committee

An independent DSMB will be established as an oversight role to monitor the overall conduct of the trial, regularly review accumulating trial data and ensure compliance with scientific, regulatory and ethical standards. The DSMB will be a multidisciplinary group composed of academics/clinicians convened yearly. During the development stage, the DSMB will ensure data integrity. When identified, omissions, inconsistencies or errors will be explored and resolved. Based on the results of the interim analyses and in accordance with the prespecified decision rules, the DSMB will make recommendations to the Internal Trial Committee (ITC) regarding the progress and conduct of the trial (ie, advising the ITC on whether each intervention arm should be maintained or dropped across interim stages, or even whether the trial should be terminated for futility). For this reason, the board members will not be blinded to allocation to enable a more comprehensive and integrated data review.

### Trial monitoring

An ITC will be established to monitor all aspects of the conduct and progress of the clinical trial, ensuring adherence to the protocol, monitoring adverse events and safeguarding participants’ safety and the trial’s quality. The ITC will be composed of the principal investigator, the trial coordinator and at least another co-investigator, convening weekly. The principal investigator will be responsible for overseeing participants’ safety and safeguarding their best interests. The principal investigator will be blinded to allocation, unless unblinding is essential to ensure the participant’s safety.

## Ethics and dissemination

### Research ethics approval

This study has been prospectively approved by the HREC of The University of Sydney (HREC Approval No. 2023/HE000772) and prospectively registered with the Australian New Zealand Clinical Trials Registry (ANZCTR) (https://www.anzctr.org.au/) (Trial Registration: ACTRN12624000319572; Registration date: 25 March 2024). A Universal Trial Number (UTN) has been generated (U1111-1301-6286). Informed consent will be obtained from all participants.

### Dissemination policy

The findings of this study will be disseminated to the scientific community through publications in relevant peer-reviewed journals, reports and presentations at scientific forums and conferences. Participants will be provided with a summary of the study findings in lay language. The study results will also be strategically disseminated to the wider community and relevant policymakers to increase awareness of evidence-based recommendations. We will seek to establish a partnership with Musculoskeletal Health Australia to share the study findings with their network of researchers and consumers, boosting the breadth and depth of our communication capabilities.

### Protocol amendments

This protocol is version number 6, dated 12 December 2024. Any changes to the study protocol will be submitted to the HREC for review and approval and acknowledged by the study sponsor prior to implementation.

### Consent or assent

Informed consent will be obtained from all participants before any study-related procedures. After reading the Participant Information Statement (online supplemental material 1), potentially eligible participants who are willing to participate in the trial and comply with the study procedures will be invited to the consent procedures via REDCap. The online signature will be collected by ticking a box and selecting ‘proceed’ on the online Participant Consent Form (online supplemental material 2).

### Confidentiality

Access to the data collected during the trial will be restricted to the principal investigator, the trial coordinator and members of the research team, according to prespecified blinding arrangements. Participants’ privacy will be protected, and data confidentiality will be maintained during all the stages of the study, for archiving and storage, and for publication and dissemination purposes. Study data and associated records will be exported and stored in a re-identifiable (coded) format on a password-protected research data storage infrastructure hosted and managed by the University of Sydney. Once data collection is completed, identifiers will be removed from the dataset to protect participants’ privacy.

### Ancillary and post-trial care

All participants will have access to specific components of the app (according to their group allocation) during the entire study (ie, 1 year after the last recruited participant completes the intervention period). After this period, we will offer offline access to the full content of the app to all participants in the study for an additional 6 weeks.

### Trial registration

This trial has been prospectively registered with the Australian New Zealand Clinical Trials Registry (ANZCTR) (http://www.anzctr.org.au/) (ACTRN12624000319572; Registration Date: 25 March 2024) prior to the enrolment of the first participant. A Universal Trial Number (UTN) has been generated (U1111-1301-6286).

### Protocol and statistical analysis plan

The full statistical analysis plan will not be published alongside this study protocol.

### Data sharing

The datasets generated and/or analysed during the current study will be available from the corresponding author on reasonable request. The datasets will not be made publicly available due to restrictions in participants’ consent regarding their data being shared by the University of Sydney.

## Supplementary material

10.1136/bmjopen-2024-098324 online supplemental file 1

10.1136/bmjopen-2024-098324 online supplemental file 2
