# Supplementary material for: My Back Exercise app: an automated exercise intervention supported by educational notifications, a sleep programme and diet advice to improve function in people with chronic non-specific low back pain – protocol for a superiority, adaptive multi-arm multi-stage randomised controlled trial
Source: BMJ Open. 2025 Aug 1;15(7):e098324. doi: 10.1136/bmjopen-2024-098324 (PMC12314989; doi:10.1136/bmjopen-2024-098324)
Supplement: online supplemental file 1 [file bmjopen-15-7-s001.pdf]

Participant Information Statement

Research Study: My Back Exercise App - An automated exercise intervention supported by educational notifications, a sleep behavioural program, and diet advice: protocol for an adaptive multi-arm multi-stage randomised controlled trial.

Prof. Paulo Ferreira (Responsible Researcher)
Faculty of Medicine and Health
Phone: [phone_number] | Email: [email_address]

1. What is this study about?

We are conducting a research study about the **"My Back Exercise App"**, a mobile health application developed for people with low back pain. The purpose of this research is to test whether the **"My Back Exercise App"** can help people with chronic low back pain to improve their physical function, as well as reduce their pain intensity and improve their quality of life. Taking part in this study is voluntary.

You have been invited to take part in this study because you are 18 years of age or older, you have had low back pain for at least 12 weeks, you have a smartphone or tablet with an Internet connection, and a sufficient understanding of English. Your contact details were obtained from the information you provided via the Pre-Screening Questionnaire, available from the study advertisements.

Please read this sheet carefully and ask questions about anything that you don't understand or want to know more about.

2. Who is running the study?

The study is being carried out by the following researchers:

- Prof Paulo Ferreira, Professor, Faculty of Medicine and Health, Sydney Musculoskeletal Health, The University of Sydney. (Principal Investigator, PhD Student Supervisor)
- MSc Anelise Moreti Cabral Silveira, PhD student, Faculty of Medicine and Health, Sydney Musculoskeletal Health, The University of Sydney. (PhD Student and Trial Coordinator)
- Dr Carlos Mesa Castrillon, Postdoctoral Research Associate, Faculty of Medicine and Health, Sydney Musculoskeletal Health, The University of Sydney. (Postdoctoral Fellow and Co-Investigator)

- MSc Josielli Comachio, PhD student, Faculty of Medicine and Health, Sydney Musculoskeletal Health, The University of Sydney. (PhD Student and Co-Investigator)
- Dr Emma Ho, Postdoctoral Research Associate, Faculty of Medicine and Health, Sydney Musculoskeletal Health, The University of Sydney. (Postdoctoral Fellow and PhD Student Supervisor)
- Dr Paula Beckenkamp, Senior Lecturer, Faculty of Medicine and Health, Sydney Musculoskeletal Health, The University of Sydney. (Co-Lead Investigator and PhD Student Supervisor)
- Katharine Roberts, PhD student, Faculty of Medicine and Health, Sydney Musculoskeletal Health, The University of Sydney. (PhD Student and Co-Investigator)
- Prof. Manuela Ferreira, Professor, Faculty of Medicine and Health, Sydney Musculoskeletal Health, The University of Sydney. (Co-Lead Investigator)
- Associate Professor Christopher Gordon, Associate Professor, Faculty of Medicine, Health and Health Sciences, Macquarie University. (Co-Lead Investigator, Sleep Expert)
- Associate Professor Serigne Lo, Associate Professor, Charles Perkins Centre, The University of Sydney. (Statistician)
- [name, affiliation, and role of operational team members]

The Chief Investigator's role in building the mobile application was to develop the content and make decisions about how this content is displayed within the app (workflows and format). An independent company (Trade eXpansion) developed the app, including its design and front-end coding.

Anelise Moreti Cabral Silveira is conducting this study as the basis for the degree of Doctor of Philosophy (Medicine and Health) at The University of Sydney.

This study is being funded by the Faculty of Medicine and Health, The University of Sydney, through the Rewarding Research Success Funds.

The researchers have no known conflicts of interest to declare.

3. Who can take part in the study?

We are seeking people aged 18 years or over who report an episode of non-specific low back pain of at least 12 weeks duration, had their low back pain diagnosed by a healthcare practitioner, have a smartphone or tablet with an Internet connection to download the app, have independent mobility and eyesight to use the app (i.e., do not regularly use mobility aids), and have a sufficient understanding of English.

You cannot take part in this study if you have known or suspected serious pathology in the spine (e.g., a bone fracture of the spine); have a specific diagnosis of LBP (e.g., sciatica, severe spinal stenosis – narrowing of the spine); have radicular symptoms (e.g., reflex changes, motor loss, nerve root pain); have had spinal surgery in the past 12 months; have LBP caused by involvement in a road traffic crash in the last 12 months; are currently receiving ongoing litigation; have fibromyalgia or systemic/inflammatory condition(s) that are not controlled (e.g., systemic lupus erythematosus, multiple sclerosis); have a co-morbid health condition(s) diagnosed by a medical practitioner that would prevent participation in physical activity or exercise programs (e.g., chronic heart problems).

Once you read this Participant Information Statement, if you decide to take part in this study, you will be asked to sign the online (electronic) **Participant Consent Form**. The consent form must be signed before any further study procedures occur. You will then be asked to **complete an Eligibility Screening Form** to assess if you are eligible to take part in the study. This procedure will occur via a phone call in one session of approximately 5 minutes. If the screening questionnaire shows that you meet the requirements, you will be able to start the research project.

4. What will the study involve for me?

After signing the consent form, you will be enrolled in the study and immediately assigned a **unique study identification (ID) code**. This code will be used on all study documents to protect your privacy. Once enrolled in the study, you will be invited to complete the initial assessment online (electronically). Once you complete your initial assessment, you will be put into a group by chance (randomly). This process will be conducted by a computer software program. There are several possible groups in this study. Each group will receive access to different combinations of components within the My Back Exercise App (i.e., education, exercise, push notifications, sleep tips, and diet tips). You have an equal chance to be assigned to one of these groups.

After being put in one of these groups, all participants will be asked to:

- **Download and install the "My Back Exercise" App on your mobile phone or tablet and utilise it**. You will receive an email from the research team containing details on how to download, install and access the app, followed up by a phone call to help with these procedures and check if the app is working properly. You will also receive a welcome email prompting you to access the app and start your intervention program. These procedures will occur online (electronically) and will last 6 weeks from the beginning of the study. The utilisation of the app will involve a time commitment of at least 30 minutes to 1 hour per day, 3 days per week, totalling at least 9 hours across the 6-week intervention period.
- **Complete study forms and questionnaires about you and your health**. You will receive an email from the research team containing a link to a secured web platform called REDCap, hosted by The University of Sydney. This procedure will occur online (electronically) in four sessions across one year: at the beginning of the study and after 6 weeks, 3 months and 12 months. The completion of all study

forms and questionnaires will involve a time commitment of about 1 hour per session, totalling 4 hours across the entire study.

- **Report adverse events**. Adverse events are undesirable, unfavourable or unintended signs or symptoms you may experience, and it is essential for us to understand. You will receive an email from the research team containing a link to a secured web platform called REDCap, hosted by The University of Sydney. This procedure will occur online (electronically) in 3 fortnightly sessions from the beginning of the study. The reporting of adverse events will involve a time commitment of about 5 minutes per session, totalling 15 minutes across the entire study.

Therefore, your involvement in the study will last for approximately 12 months.

5. Can I withdraw once I've started?

Being in this study is completely voluntary and you can withdraw from the study at any time. Your decision will not affect your current or future relationship with the researchers or anyone else at The University of Sydney.

If you decide to take part in the study and then change your mind, you can withdraw at any time up until the data is analysed. Before you withdraw, please notify a member of the research team by email. A member of the research team will inform you if there are any special requirements linked to withdrawing. If you do withdraw, you will be asked to complete and sign a "Revocation of Consent Form", provided to you by the research team.

Once you withdraw, we will not collect any more information from you. Any information that we have already collected however will be kept in our study records and may be included in the study results. If you do not want your data to be included, you must tell the researchers at the time you withdraw from the research project.

6. Are there any risks or costs?

This study has been designed to minimise or prevent potential risks. Nevertheless, adverse events (undesirable, unfavourable or unintended signs or symptoms) are expected and may include flare-ups of low back pain, exercise-induced muscle pain, soreness and cramps, and unexpected trips and falls.

You can contact the research team directly to report these events. In addition, you will receive fortnightly emails reminding you to report them during the intervention period. The research team will monitor reported adverse events until their resolution. Potential harms resulting from participation in the study will not incur any special compensation arrangements.

Aside from giving up your time, we do not expect that there will be any costs associated with participating in this study. Therefore, no financial incentives will be offered.

7. What if injury or complications happen?

In the unlikely event of injuries or complications as a result of this study, you should contact the research team as soon as possible. They will assist you in advising appropriate management strategies. Potential harms resulting from participation in the study will not incur any special compensation arrangements.

8. What happens when the study ends?

All participants will have access to specific components of the app (according to their group) during the entire study (i.e., 1 year after the last recruited participant completes the intervention period). After this period, we will offer offline access to the full content of the app to all participants in the study for an additional 6 weeks.

9. Are there any benefits?

You will not receive any **direct benefit** from participating in this study.

10. What will happen to the information that is collected?

By providing your consent, you are agreeing to us collecting information about you for the purposes of this study.

Data collection will be performed via the Research Electronic Data Capture (REDCap), a secure web application for building and managing online surveys and databases, supported by the University of Sydney. Additionally, basic interactions with the mobile application will be automatically collected, including which, how often and how long each page was accessed.

Any information you provide us, including confidential data that can reveal personal information from you, will be stored securely during and after the study on the Research Data Store (RDS), a password-protected research data storage infrastructure hosted and managed by the University of Sydney. Access to this information will be restricted to the research team conducting the study.

Your privacy will be protected, and data confidentiality will be maintained during all the stages of the study, for archiving and storage, and for publication and dissemination purposes. Identifiable information will only be disclosed with your permission unless we are required by law to disclose material. We anticipate study findings will be published. You will not be individually identifiable in these publications.

Study records will be retained for a minimum of 15 years after study completion or after the last study publication, in accordance with current regulatory standards for intervention studies established by the National Statement on Ethical Conduct in Human Research and the Australian Code for the Responsible Conduct of Research.

We will keep the information we collect for this study, and we may use it in future research. By providing your consent you are allowing us to use your information in future

projects. We will seek ethical approval before using the information in these future projects.

11. Will I be told the results of the study?

You have a right to receive feedback about the overall results of this study. You can indicate you are interested in receiving feedback by providing your contact details on the consent form and answering the relevant questions. This feedback will be in the form of a brief lay summary.

12. What if I would like further information?

When you have read this information, the following researcher/s will be available to discuss it with you further and answer any questions you may have:

- Anelise Moreti Cabral Silveira, PhD student:
  Email: [email_address]; Mobile: [phone_number].

- Dr Paula Beckenkamp, Senior Lecturer:
  Email: [email_address]; Telephone: [phone_number].

13. What if I have a complaint or any concerns?

The ethical aspects of this study have been approved by the Human Research Ethics Committee (HREC) of The University of Sydney (HREC Approval No. 2023/HE000772) according to the *National Statement on Ethical Conduct in Human Research (2007).*

If you are concerned about the way this study is being conducted or wish to make a complaint to someone independent from the study, please contact the University:

Human Ethics Manager
[email_address]
[phone_number]

This information sheet is for you to keep.
