# Supplementary material for: My Back Exercise app: an automated exercise intervention supported by educational notifications, a sleep programme and diet advice to improve function in people with chronic non-specific low back pain – protocol for a superiority, adaptive multi-arm multi-stage randomised controlled trial
Source: BMJ Open. 2025 Aug 1;15(7):e098324. doi: 10.1136/bmjopen-2024-098324 (PMC12314989; doi:10.1136/bmjopen-2024-098324)
Supplement: online supplemental file 2 [file bmjopen-15-7-s002.pdf]

Participant Consent Form

THE UNIVERSITY OF SYDNEY

Research Study: My Back Exercise App - An automated exercise intervention supported by educational notifications, a sleep behavioural program, and diet advice: protocol for an adaptive multi-arm multi-stage randomised controlled trial.

Prof. Paulo Ferreira (Responsible Researcher)
Faculty of Medicine and Health
Phone: [phone_number] | Email: [email_address]

Participant Name ______________________________

I agree to take part in this research study. In giving my consent, I confirm that:

- The details of my involvement have been explained to me, and I have been provided with a written Participant Information Statement to keep.
- I understand the purpose of the study is to investigate whether the **"My Back Exercise App"** can help people with chronic low back pain improve their physical function, as well as reduce their pain intensity and improve their quality of life.
- I acknowledge that the risks and benefits of participating in this study have been explained to me to my satisfaction.
- I understand that in this study I will be required to download and install the "My Back Exercise" App on my mobile phone or tablet and utilise it for 6 weeks; complete study forms and questionnaires about me and my health at the beginning of the study and after 6 weeks, 3 months and 12 months; and report adverse events in 3 fortnightly sessions.
- I understand that my information may be used in future research projects.
- I understand that being in this study is completely voluntary.
- I am assured that my decision to participate will not have any impact on my relationship with the research team or the University of Sydney.
- I understand that I am free to withdraw from this study at any time and that I can choose to withdraw any information I have already provided (unless the data has already been de-identified or analysed).

- I have been informed that the confidentiality of the information I provide will be protected and will only be used for purposes that I have agreed to. I understand that information identifying me will only be told to others with my permission, except as required by law.

- I understand that the results of this study may be published, and that publications will not contain my name or any identifiable information about me.

- I confirm the following:

**I consent to my data being used in future research**	Yes ❑	No ❑
**I would like feedback on the overall results of this study**	Yes ❑	No ❑
**I would like to hear about future study opportunities**	Yes ❑	No ❑

If you answered **yes**, please provide your preferred contact details (email/telephone/postal address):

___

___

___

- I understand that after I sign and return this consent form it will be retained by the research team and that I may request a copy at any time.

Participant Name ______________________

Date ______________________

Participant Signature ❑ Tick this box if you wish to sign the Participant Consent Form.

The signature is provided by ticking the box and clicking "proceed".
