# Supplementary material for: My Back Exercise app: an automated exercise intervention supported by educational notifications, a sleep programme and diet advice to improve function in people with chronic non-specific low back pain – protocol for a superiority, adaptive multi-arm multi-stage randomised controlled trial
Source: BMJ Open. 2025 Aug 1;15(7):e098324. doi: 10.1136/bmjopen-2024-098324 (PMC12314989; doi:10.1136/bmjopen-2024-098324)
Supplement: Supplementary file 3 [file bmjopen-2024-098324.reviewer_comments.pdf]

PEER REVIEW HISTORY

BMJ Open publishes all reviews undertaken for accepted manuscripts. Reviewers are asked to complete a checklist review form and are provided with free text boxes to elaborate on their assessment. These free text comments are reproduced below.

ARTICLE DETAILS

Title (Provisional)

My Back Exercise App - An automated exercise intervention supported by educational notifications, a sleep program, and diet advice to improve function in people with chronic non-specific low back pain: Protocol for a superiority, adaptive multi-arm multi-stage randomised controlled trial

Authors

Silveira, Anelise Moreti Cabral; Comachio, Josielli; Mesa Castrillon, Carlos Ivan; Roberts, Katharine; Beckenkamp, Paula; Ho, Emma K; Ferreira, Manuela; Gordon, Christopher James J; Lo, Serigne; Bennell, Kim L; Nelligan, Rachel K; Field, Rowena J; Ferreira, Paulo

---

VERSION 1 - REVIEW

**Reviewer**	**1**
**Name**	**Wood, Lianne**
**Affiliation**	**University of Exeter**
**Date**	**28-Apr-2025**
**COI**	**None**

Dear Authors

Well done on a well written paper and protocol. This trial proposes some interesting and novel concepts that will further the field of CLBP management.

I know that digital interventions are widely accepted and used for RCT design in Australia, but I did wonder what proportion of the population has access to smart phones? Does this represent the majority (>80%) of the population or are there still a proportion of people without access to smart phones/ internet access. Do you know how many people in Australia this represents and therefore what proportion are automatically excluded from participating in this study? I don't envisage it to be large proportion of the population, but I think it important from an Equality and Inclusivity perspective to describe how many people may be excluded from the study as a result.

In addition, I was not clear how participants would be notified about their allocation. I can see that the randomisation schedule is performed electronically, and that the person

determining eligibility is unaware of allocation, but I was not clear how the participant would be notified. Will there be a third party checking that allocation and app components offered are accurate? Even if this was for the first 10-20% it may be important to check that the allocation occurs as planned.

In the secondary analyses proposed, will there be any scope to assess the effect of adherence? You specify acceptable adherence thresholds to exercise in particular, but also mention education and diet (without thresholds) but it is not clear what the implications will be if participants do not meet these thresholds. Will you compare the differences between the EARS scale and the thresholds of engagement you have set?

**Reviewer**	**2**
**Name**	**Tsang, Sharon**
**Affiliation**	**The Hong Kong Polytechnic University, Rehabilitation Sciences**
**Date**	**07-May-2025**
**COI**	**None**

Thank you for inviting me to review this novel trial which examines the efficacy of an tailored-developed app that integrates education, notifications, exercise, sleep and diet modules on clinical outcomes amongst those with chronic nonspecific low back pain. The protocol is overall comprehensive, clear and organised. There are a few points listed below for consideration to improve the clarity and justification of the present protocol.

Clarification and more details would be needed for the followings:

- Procedures related to verification of the individuals who are the genuine participants that fulfill all the inclusion criteria and free from any of the listed exclusion criteria listed in this trial.

- Provide more details/justification related to the pre-specified decsion rules (in addition to those included in the interim analysis section of this version), which is planned at the end of the first stage for deciion making on dropping of the chosen arm(s) of this RCT, in particular with the evidence of minimum duration of respective trial components to show their effect on the listed outcome measures.

- With reference to the specified objectives in evaluating the impact of this trial multiple domains (specified as improving the self-care and pain coping strategies, page 19), please consider re-organise the list of secondary outcomes to explicitly present them according to the respective domain each of them address.

VERSION 1 - AUTHOR RESPONSE

Reviewer 1: Prof. Lianne Wood, University of Exeter

Comments to the Author:

Dear Authors

Well done on a well written paper and protocol.

1. **This trial proposes some interesting and novel concepts that will further the field of CLBP management. I know that digital interventions are widely accepted and used for RCT design in Australia, but I did wonder what proportion of the population has access to smart phones? Does this represent the majority (>80%) of the population or are there still a proportion of people without access to smart phones/ internet access. Do you know how many people in Australia this represents and therefore what proportion are automatically excluded from participating in this study? I don't envisage it to be large proportion of the population, but I think it important from an Equality and Inclusivity perspective to describe how many people may be excluded from the study as a result.**
   <u>*Authors' Response*</u>*: We thank the reviewer for the insightful comment. We have added, to the 'Recruitment' section of the manuscript, a paragraph to address the concerns related to smartphone and Internet access in the Australian context, as follows:*
   "The digital divide was an additional layer of complexity that was carefully considered from an equity and inclusivity perspective when designing the study. In the Australian context, critical barriers to digital inclusion include uneven access to networks and digital infrastructure, affordability of devices and data, and a lack of digital literacy and ability, as measured by The Australian Digital Inclusion Index (ADII) [93]. In 2017, Australia was one of the leading adopters of smartphones globally, with 88% of Australians owning one [94]. In 2023, 99% of Australian adults had Internet access, with 95% of online adults connecting mostly over their mobile phones [95]. Finally, the 2023 Australian National ADII score was 73.2, with 14.2% and 9.4% Australians classified as digitally excluded and highly digitally excluded, respectively [93]. Although we do not envisage that a large proportion of the population will be automatically excluded from participating in this study due to digital inequalities, we recognise that this may pose a limitation of the study, with potential impact on recruitment and attrition rates, as well as adherence to the intervention." (Page 32, lines 1-13).

2. **In addition, I was not clear how participants would be notified about their allocation. I can see that the randomisation schedule is performed electronically, and that the person determining eligibility is unaware of allocation, but I was not clear how the participant would be notified. Will there be a third party checking that allocation and**

app components offered are accurate? Even if this was for the first 10-20% it may be important to check that the allocation occurs as planned.

Authors' Response: We thank the reviewer for this comment. The manuscript was modified to clarify the allocation procedures.

"After randomisation, participants will receive an email containing detailed instructions on how to download, install, and access the app. This will be followed by a phone call from a member of the research team to help with these procedures if needed, ensure proper functioning of the app, and confirm participants only have access to the app components they have been randomly allocated to." (Page 33, lines 13-20).

3. **In the secondary analyses proposed, will there be any scope to assess the effect of adherence? You specify acceptable adherence thresholds to exercise in particular, but also mention education and diet (without thresholds) but it is not clear what the implications will be if participants do not meet these thresholds. Will you compare the differences between the EARS scale and the thresholds of engagement you have set?**

   Authors' Response: We thank the reviewer for this comment. The section describing procedures for monitoring adherence to the intervention has been expanded, with the inclusion of adherence assessment methods and adherence thresholds for all intervention components (i.e., exercises, education, sleep, and diet), as well as plans for statistical analyses, as follows:

   "To measure adherence to the intervention, participants will complete a self-reported activity tracker within the My Back Exercise app for the exercise and sleep modules (which offer tailored content), and a weekly electronic logbook via REDCap for the education and diet modules. The invitation to complete the weekly electronic logbook will be sent via scheduled and automated emails via REDCap. Additionally, app use and engagement will be collected in a de-identified format using data analytics, drawn from the app's backend system (e.g., frequency of use, time spent, content accessed) and using the Matomo web analytics platform [75]. At six and nine months after randomisation, there will also be a brief phone call with the participants to follow up on adherence and engagement with the app. Adherence will be calculated for each study arm and the entire study, as described below.

   For the exercise module, participants will be recommended to complete at least three exercise sessions per week, totalling 18 sessions over the 6-week exercise program. Data will be drawn primarily from the app's self-reported daily activity tracker, and additional insights will be provided by data analytics from Matomo [75]. Adherence levels will be assessed based on the proportion of self-reported completed exercise sessions relative to the number of recommended sessions and categorised as low (0–35%, or up to six exercise sessions completed), moderate (35–70%, or seven to 12 exercise sessions completed), and high (70–100%, or 13 to 18 exercise sessions completed). Adherence will be considered acceptable when participants complete at least 70% of their prescribed exercise program [76].

   For the sleep module, participants may choose weekly or daily goals over the 4-week sleep program. Data will be drawn primarily from the app's self-reported daily or weekly activity

tracker according to the goal selected by the participant, and additional insights will be provided by data analytics from Matomo [75]. Adherence levels will be assessed based on the proportion of self-reported completed goals relative to the number of recommended goals and categorised as low (0–35%), moderate (35–70%), and high (70–100%). Adherence will be considered acceptable when participants complete at least 70% of their prescribed sleep program.

For the education and diet components, participants will be recommended six structured modules over the 6-week app program. Data will be drawn primarily from the weekly electronic logbooks, categorised as "not started", "partially completed", or "completed", and additional insights will be provided by data analytics from Matomo [75]. Adherence levels will be assessed based on the number of self-reported completed modules and categorised as low (0–35%, or up to two modules completed), moderate (35–70%, or three to four modules completed), and high (70–100%, or five to six modules completed). Adherence will be considered acceptable when participants complete at least 70% of their prescribed education or diet programs.

A planned exploratory analysis will be conducted to investigate the potential impact of adherence on the magnitude of the treatment effects." (Page 20, lines 3-25; Page 21, lines 1-15).

Reviewer 2: Dr. Sharon Tsang, The Hong Kong Polytechnic University

Comments to the Author:

Thank you for inviting me to review this novel trial which examines the efficacy of an tailored-developed app that integrates education, notifications, exercise, sleep and diet modules on clinical outcomes amongst those with chronic nonspecific low back pain. The protocol is overall comprehensive, clear and organised. There are a few points listed below for consideration to improve the clarity and justification of the present protocol.

Clarification and more details would be needed for the followings:

4. **Procedures related to verification of the individuals who are the genuine participants that fulfill all the inclusion criteria and free from any of the listed exclusion criteria listed in this trial.**

   <u>Authors' Response</u>: *We thank the reviewer for this comment. The procedures to verify the fulfilment of all the eligibility criteria have been described in more detail, and more context has been provided on the methods implemented to ensure the authenticity of study respondents, as follows:*

   "Consenting participants will receive a phone call from a trained member of the research team, who will complete the Eligibility Screening Form over the phone and determine eligibility. Data will be entered directly into the Research Electronic Data Capture (REDCap). The phone call will also be essential to verify the authenticity of responses and

ensure the individuals are genuine participants. To be included in the study, participants will need to meet all the inclusion criteria and none of the exclusion criteria." (Page 12, lines 10-15).

"Additional Exercise and Physical Activity Screening Questions will be applied and considered on a case-by-case basis to determine if medical clearance for participation in an unsupervised, home-based exercise program is required before enrolment. In that case, the participant will be given a Medical Clearance Referral Form to be completed by a medical practitioner (e.g., general practitioner)." (Page 13, lines 18-22).

"The welcome page will be secured by a Google reCAPTCHA feature, enabled to help protect the public survey from abuse by 'bots' and prevent automated software programs from submitting fraudulent responses. After passing the reCAPTCHA test, participants will have access to the welcome page and the Pre-Screening Questionnaire." (Page 31, lines-17-20).

5. **Provide more details/justification related to the pre-specified decision rules (in addition to those included in the interim analysis section of this version), which is planned at the end of the first stage for decision making on dropping of the chosen arm(s) of this RCT, in particular with the evidence of minimum duration of respective trial components to show their effect on the listed outcome measures.**
   Authors' Response: We thank the reviewer for this comment. More details and justification related to the pre-specified decision rules have been provided, as follows:
   "The decision rules were carefully designed to minimise the risk of prematurely and incorrectly dropping promising treatment arms, potentially leading to a failure to reject the null hypothesis (Type II error), while ensuring that the pre-planned adaptations would still guarantee increased trial efficiency. Two complementary decision rules were proposed to account for the comparison between each treatment arm against the control group (first rule) and the pairwise comparison among all treatment arms (second rule). Both decision rules are intended to work as indicators of futility, therefore configuring a "drop-the-loser" design that also allows early stopping for futility. Early stopping for efficacy was not planned in this trial." (Page 37, lines 1-8).

6. **With reference to the specified objectives in evaluating the impact of this trial multiple domains (specified as improving the self-care and pain coping strategies, page 19), please consider re-organise the list of secondary outcomes to explicitly present them according to the respective domain each of them address.**
   Authors' Response: We thank the reviewer for this comment. The secondary outcomes have been reorganised into domains for clarity, as follows (Page 22, line 9, to Page 25, line 16):

   "Symptoms and function:
   - *Pain intensity.*
   - *Pain frequency.*
   - *Disability.*

Quality of life and general health:
- *Health-related quality of life (HRQoL).*
- *Sleep quality.*
- *Consumption of highly processed food.*

Health behaviours and beliefs:
- *Exercise importance.*
- *Exercise self-efficacy.*
- *Adherence to exercise recommendations.*

Healthcare utilisation and engagement:
- *Co-intervention use.*
- *Health literacy level.*

Patient perception of change:
- *Global perceived effect.*
- *Impression of change in overall status.*

Satisfaction and usability of the app:
- *Overall satisfaction.*
- *Usefulness of the appl.*
- *Frequency of the push notifications.*

Process Measures:
- *Conversion rate.*
- *Recruitment rate.*
- *Rate of completed follow-up.*

Other Measures:
- *Smallest Worthwhile Effect (SWE).*
- *Adverse Events."*

VERSION 2 - REVIEW

**Reviewer**	**1**
**Name**	**Wood, Lianne**
**Affiliation**	**University of Exeter**
**Date**	**27-Jun-2025**
**COI**	

Dear Author team,

Thank you for your thorough and comprehensive responses to my and the other reviewers' comments. I have no further comments and think this is a high quality protocol suitable for publication.

**Reviewer**	**2**
**Name**	**Tsang, Sharon**
**Affiliation**	**The Hong Kong Polytechnic University, Rehabilitation Sciences**
**Date**	**25-Jun-2025**
**COI**	

Thanks for the comprehenive revision made by the authors for which they have already addressed all concerns from the previous round of review by providing sufficient details in highly organised manner, except for one outstanding issue with some of the in-text citations. There are still in-text citation errors found in the tracked version (pages 21, 32 and 39) and clean version (pages 27 and 33) for some references included in this revised version.

Suggest to add "detailed in Section 2.5" for sentence (line 7-8, page 30 of the tracked version) for cross-referencing the list of self-report questionnaires used in this study for collecting patient-reported outcomes.

VERSION 2 - AUTHOR RESPONSE

Reviewer 1: Prof. Lianne Wood, University of Exeter**.**

Comments to the Author:

7. **Dear Author team,**
   Thank you for your thorough and comprehensive responses to my and the other reviewers' comments. I have no further comments and think this is a high quality protocol suitable for publication.
   Authors' Response: We thank the reviewer for this comment and appreciate the time and effort dedicated to providing feedback on our manuscript.

Reviewer 2: Dr. Sharon Tsang, The Hong Kong Polytechnic University.

Comments to the Author:

1. **Thanks for the comprehenive revision made by the authors for which they have already addressed all concerns from the previous round of review by providing sufficient details in highly organised manner, except for one outstanding issue with some of the in-text citations. There are still in-text citation errors found in the tracked version (pages 21, 32 and 39) and clean version (pages 27 and 33) for some references included in this revised version.**
   Authors' Response: *We apologise for the inconvenience and thank the reviewer for providing the page numbers where the errors persisted, as this helped us identify that the errors were related to cross-reference links to figures and tables. We confirm that the cross-references were converted to plain text throughout the manuscript (Page 19, line 12; Page 28, lines 1-2; Page 29, line 1; Page 33, line 3; Page 51, lines 1-3).*

2. **Suggest to add "detailed in Section 2.5" for sentence (line 7-8, page 30 of the tracked version) for cross-referencing the list of self-report questionnaires used in this study for collecting patient-reported outcomes.**
   Authors' Response: *We thank the reviewer for the suggestion and confirm that we added "detailed in Section 2.5" for cross-referencing the list of self-report questionnaires used in the study (Page 26, line 10). We have also rearranged the outcome measures listed in the "Objectives" section (Page 10, lines 24-25 – Page 11, lines 1-4) and Table 1 (Page 29-30), to reflect the order in which they are described in the text.*
